# Measurement of tibial slope angle after medial opening wedge high tibial osteotomy: case series

**DOI:** 10.1590/S1516-31802009000100008

**Published:** 2009-05-11

**Authors:** Ricardo Hideki Yanasse, Carlos Eduardo Cavallari, Felipe Lourenço Chaud, Arnaldo José Hernandez, Roberto Ryuiti Mizobuchi, Marcos Henrique Laraya

**Affiliations:** 1 MD. Attending physician in the Sports Medicine Group, Institute of Orthopedics and Traumatology, Hospital das Clínicas, School of Medicine, Universidade de São Paulo, São Paulo, Brazil.; 2 MD. Attending physician in the Knee Group, Faculdade de Medicina de Marília (Famema), Marília, São Paulo, Brazil.; 3 MD. Attending physician in the Shoulder Group, Institute of Orthopedics and Traumatology, Hospital das Clínicas, School of Medicine, Universidade de São Paulo, São Paulo, Brazil.; 4 MD, PhD. Head of the Sports Medicine Group, Institute of Orthopedics and Traumatology, Hospital das Clínicas, School of Medicine, Universidade de São Paulo, São Paulo, Brazil.; 5 MD. Head of the Discipline of Orthopedics and Traumatology, Faculdade de Medicina de Marília (Famema), Marília, São Paulo, Brazil.; 6 MD, MSc. Former attending physician in the Knee Surgery Group, Institute of Orthopedics and Traumatology, Hospital das Clínicas, School of Medicine, Universidade de São Paulo, São Paulo, Brazil.

**Keywords:** Knee, Osteoarthritis, Tibia, Osteotomy, Radiography., Joelho, Osteoartrite, Tíbia, Osteotomia, Radiografia.

## Abstract

**CONTEXT AND OBJECTIVE:**

In the past, changes in tibial slope were not considered when planning or evaluating osteotomies, and success in high tibial osteotomy was related to the alignment and amount of femorotibial angular correction. The aim here was to measure changes in tibial slope after medial opening wedge tibial osteotomy and investigate the effect of tibial slope angle on the clinical results.

**DESIGN AND SETTING:**

Retrospective review study on a series of cases, at the Department of Orthopedics and Traumatology, Faculdade de Medicina de Marília (Famema), Marília, Brazil.

**METHODS:**

Twenty-eight patients were studied, and a total of thirty-one knees. Lateral roentgenograms of the tibia were used pre and postoperatively to measure the tibial slope based on the proximal tibial anatomical axis. The clinical results were measured using the Lysholm knee score.

**RESULTS:**

There was an average increase in tibial slope angle after surgery of 2.38° (95% confidence interval: ± 0.73°). There was no correlation (r = -0.28) between the postoperative Lysholm knee score and the difference in tibial slope angle from before to after surgery (P = 0.13).

**CONCLUSION:**

Medial opening wedge tibial osteotomy led to a small increase in tibial slope. No significant correlation was found between increased tibial slope and short-term clinical results after high tibial osteotomy. Other clinical studies are needed in order to establish whether extension or flexion osteotomy could benefit patients with medial compartment gonarthrosis.

## RESUMO

**CONTEXTO E OBJETIVO:**

No passado, alterações no ângulo de inclinação ântero-posterior do planalto tibial (AIAPPT), conhecido como “slope”, não eram levadas em consideração no planejamento e avaliação das osteotomias, e o sucesso da osteotomia proximal tibial era relacionado ao grau de correção do ângulo femoro-tibial e ao alinhamento obtido. O objetivo foi mensurar a alteração no AIAPPT após a osteotomia proximal de abertura de cunha medial da tíbia e verificar o efeito da variação angular do AIAPPT sobre os resultados clínicos.

**TIPO DE ESTUDO E LOCAL:**

Estudo retrospectivo de série de casos, realizado no Departamento de Ortopedia e Traumatologia da Faculdade de Medicina de Marília (Famema), Marília, São Paulo, Brasil.

**MÉTODOS:**

Foram incluídos 28 pacientes, e um total de 31 joelhos. Foi mensurado o AIAPPT baseado no eixo anatômico proximal tibial na radiografia lateral da tíbia no pré e no pós-operatório. A avaliação clínica foi realizada através do escore de Lysholm para joelho.

**RESULTADOS:**

Houve um aumento do AIAPPT após a cirurgia em média de 2,38° (± 0,73°; intervalo de confiança, IC 95%). Não houve correlação (r = -0,28) entre os resultados obtidos pelo escore de Lysholm pós-operatório e a diferença do AIAPPT pós e pré-operatório (P = 0,13).

**CONCLUSÃO:**

A osteotomia valgizante da tíbia proximal com cunha de abertura medial levou a um pequeno aumento no AIAPPT. Não houve correlação entre o aumento do slope tibial e os resultados clínicos a curto prazo após a osteotomia proximal da tíbia. Outros estudos clínicos são necessários para comprovar se uma osteotomia de extensão ou de flexão pode trazer benefício ao paciente com artrose unicompartimental do joelho.

## INTRODUCTION

High tibial osteotomy was initially reported by Jackson and Waugh^1^ in 1961 and later popularized by the research staff of Coventry et al.^2^^,^^3^^,^^4^^,^^5^ Young patients with symptomatic medial compartment varus gonarthrosis have been treated with relative success on a long-term basis.^5^^,^^6^^,^^7^^,^^8^^,^^9^^,^^10^ Tibial osteotomy is best indicated for young patients with early osteoarthritic changes, good range of motion and no ligamentous laxity.^11^^,^^12^ However, significant relief can be obtained for active patients over the age of 60 with initial patellofemoral osteoarthritis who can sustain at least 70° of motion.^5^^,^^11^^,^^12^^,^^13^

The success achieved through high tibial osteotomy has been related to the alignment and amount of femorotibial angular correction obtained postoperatively.^2^^,^^11^^,^^14^^,^^15^^,^^16^^,^^17^^,^^18^ Coventry and Bowman recommended a valgus position from 10 to 13°.^19^ Insall et al. stated that the postoperative femorotibial angle should be between 10 and 14°, and that non-achievement of the best alignment is not the main factor leading to deterioration of the results with time.^6^^,^^17^ Saggin et al. reported better clinical results when the femorotibial angle was corrected to 6 to 14° of valgus.^17^ Hernandez et al. used the mechanical axis of the knee as a parameter, such that the axis should be located at between 60 and 70% of the tibial plateau extension, slightly laterally to the center of the knee.^20^

There has been some disagreement concerning the prognosis for osteotomy performed in the presence of patellofemoral osteoarthritis. Coventry and Insall et al. stated that the presence of patellofemoral osteoarthritis had little influence on the prognosis;^2^^,^^6^ however, other authors have stated the opposite.^11^^,^^17^^,^^21^

Several authors have reported symptom relief, of about 80 to 90%, lasting at least four years after tibial osteotomy for varus gonarthrosis.^11^^,^^15^^,^^16^^,^^17^^,^^18^ After a mean follow-up of eight years, however, only 50 to 60% of the knees still had either an excellent or a good result. ^15^^,^^16^^,^^17^^,^^18^^,^^19^

In the past, changes in tibial slope were not considered when planning or evaluating osteotomies.^22^ Recently, however, special attention has been given to the sagittal plane, since it has been observed that correction in the coronal plane of the knee simultaneously alters the tibial slope.^23^^,^^24^^,^^25^^,^^26^^,^^27^^,^^28^

## OBJECTIVE

The purpose of this study was to measure changes in tibial slope after medial opening wedge tibial osteotomy and to investigate the effect of tibial slope angle on the clinical results.

## METHODS

This study was conducted as a retrospective review on a consecutive series of patients. Proximal opening wedge tibial osteotomy was performed on a total of 41 patients between 2000 and 2006. Twenty-eight patients were available for evaluation (14 men and 17 women) with a mean age of 56 years (range, 42 to 68 years) and follow-up records of 15 to 75 months (40.8 months on average). The remaining patients were lost from follow-up or were known to have moved and changed address or died. Three patients were treated for both knees, thus making a total of 31 knees. There were 15 right knees and 16 left knees. All knees had adequate pre and postoperative radiographic imaging.

This investigation was approved by the ethics committees of the institution involved. After the patients had been fully informed about the procedures of the study, those who wished to participate signed the appropriate informed consent form.

Inclusion criteria

All the knees examined had varus deformity, at least 70° of range of motion and symptoms and radiographic signs of early medial unicompartmental gonarthrosis. The patients were all active. All knees were examined clinically to rule out anterior instability (relating to the anterior cruciate ligament, ACL), through a combination of manual tests (Lachmann, anterior drawer and Hughston’s jerk test), and to rule out posterior instability (relating to the posterior cruciate ligament, PCL).

### Surgical technique

**Figure 1.**
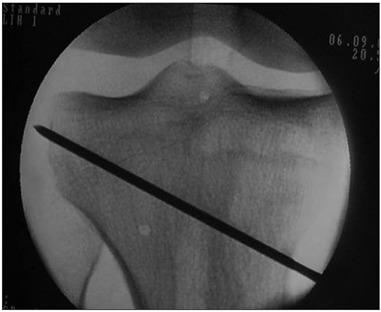
Kirschner wire acting as a guide for the osteotomy cut.

An incision was made from the medial border of the patellar tendon distally. The pes anserinus tendons were divided at their insertions and deflected posteriorly. Two Kirschner wires were placed as an osteotomy guide, entering 40 mm distally to the medial plateau and ending 10 mm from the lateral plateau (Figure 1).

**Figure 2.**
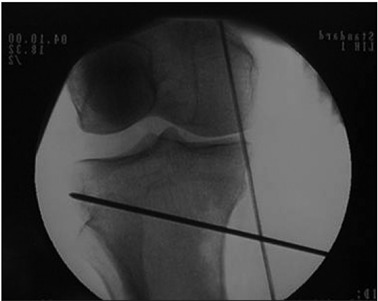
Line of the mechanical axis of the knee, crossing medially.

The size of the wedge to be added was decided in accordance with the mechanical axis of the knee. A line marked out by a metallic wire, representing the mechanical axis, was brought into view using an image intensifier, from the center of the femoral head to the center of the ankle, with the patella facing up (Figure 2).

**Figure 3.**
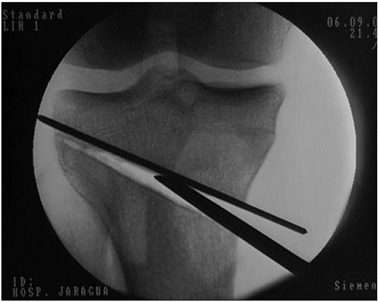
Opening of wedge, taking care not to fracture the lateral cortex.

A saw blade was used to initially mark out the depth of the tibial bone osteotomy, by gliding the blade along the Kirschner wires. The mark was made at a distance of 1 cm from the lateral cortex, using chisels. The tibia was wedged open, while taking care not to fracture the lateral cortex (Figure 3).

**Figure 4.**
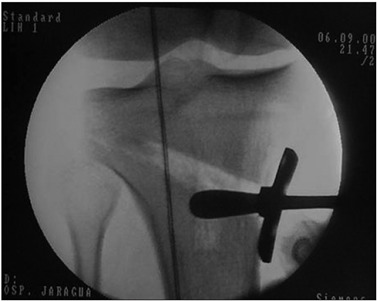
Line of the mechanical axis of the knee, crossing 60 to 70% of the plateau extent laterally.

The line marked out with the wire, representing the mechanical axis of the knee that had been seen crossing the tibial plateau medially before performing the osteotomy was now expected to cross 60 to 70% of the whole width of the tibial plateau, from the medial border (0%) to the lateral border (100%), slightly laterally to the center of the knee^20^^,^^21^ (Figure 4).

**Figure 5.**
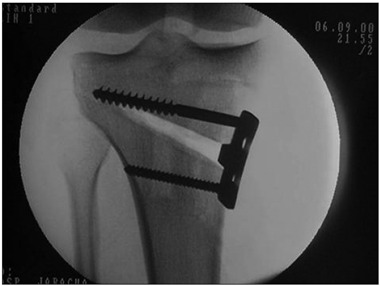
Osteotomy fixed with plate and one cancellous screw superiorly and one cortical screw inferiorly.

The gap was filled with tricortical iliac crest grafts if a plate greater than 7.5 mm was used to maintain the correction. A plate with a rectangular inset and with cancellous screws proximally and cortical screws distally was used for fixation (Figure 5). The pes anserinus was repaired and the wound closed.

### Radiographic evaluation

The radiological assessment consisted of unipedal standing radiographs in the anteroposterior and true lateral positions, made preoperatively and postoperatively. The anteroposterior radiographs were used to measure the femorotibial angle, and the lateral, to measure the posterior slope of the tibia.

**Figure 6.**
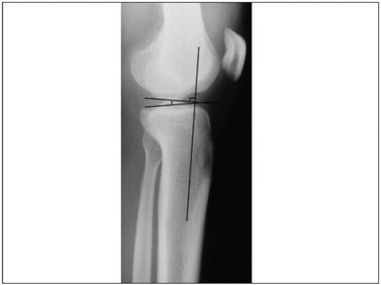
Tibial slope angle based on the tibial proximal anatomical axis.

The tibial slope was measured using the method based on the proximal tibial anatomical axis.^29^^,^^30^ The diaphyseal axis of the tibia was obtained from two points equidistant between the anterior and posterior borders of the tibia: one just below the anterior tibial tubercle and the other 10 cm distally to it. A reference line at the level of the femorotibial joint was drawn perpendicularly to the diaphyseal axis line. Another line was drawn using the highest two points of the anterior and posterior edges of the medial plateau. The angle between this line and the reference line is defined as the tibial slope^30^ (Figure 6).

### Clinical evaluation

The patients were functionally evaluated pre and postoperatively by means of the Lysholm knee score. This instrument has been translated and culturally adapted for Brazilian Portuguese and its reproducibility has been validated for patients with meniscal lesions, ACL deficiency, chondromalacia or knee arthrosis.^31^ The range of motion was measured pre and postoperatively using a standard goniometer.

In addition, a subjective grade between 0 and 10 was given by the patients according to their satisfaction with the surgery.

### Statistical analysis

The statistical analysis was carried out using the Statistica 6.0 software (Stat Soft, Inc.). Student’s t test was used for paired samples, and the Mann-Whitney nonparametric test^32^ was used when the data did not present normal distribution according to the Kolmogorov-Smirnov test. Pearson’s coefficient was used to evaluate the correlation between pairs of variables. The significance level of 5% was adopted for all statistical analyses.

## RESULTS

The mean preoperative extension deficit was 2.42° (range, 0° to 30°) and the mean postoperative extension deficit was 0.42° (range, 0° to 5°). The mean preoperative range of motion was 119° (range, 70° to 150°) and the mean postoperative range of motion was 125° (range, 80° to 150°).

The mean preoperative Lysholm knee score was 51 (range, 22 to 79), and the mean postoperative score was 90 (range, 58 to 100). The patients’ subjective satisfaction on a scale from 0 to 10 was on average 8.6 (range, 3 to 10).

**Figure 7.**
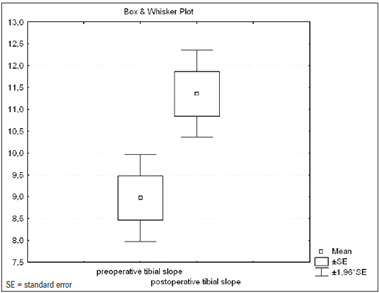
Box and whisker plot of tibial slope before and after surgery (95% confidence interval).

The mean preoperative and postoperative tibial slope angles and standard deviations (± SD) were 8.96° ± 2.83° and 11.35° ± 2.83° respectively (Figure 7). There was a significant increase in the tibial slope angle, on average 2.38^°^ (95% confidence interval, CI: ± 0.73°; SD ± 1.97°) after surgery (P = 2 x 10^-7^).

The correlation between the differences in femorotibial angle and tibial slope from before to after the operation was not statistically significant (r = 0.02; P = 0.9).

There was no significant correlation (r = -0.27) between the difference in degree of slope from before to after the operation and the patients’ postoperative Lysholm score (P = 0.13).

The correlation between age and postoperative Lysholm score was not statistically significant (r = 0.13; P = 0.48).

The correlation between the differences in degree of slope and knee extension from before to after the operation was not statistically significant (r = 0.01; P = 0.93).

There were two cases of superficial wound infection, which were treated successfully with antibiotic therapy; one of these case also presented dehiscence. One patient had pulmonary embolism that was treated with anticoagulation therapy, with full recovery. Delayed union was observed in one patient. One patient presented plate loosening, but his osteotomy had already united and the plate and screws were therefore removed.

## DISCUSSION

Several methods for measuring the tibial slope have been described.^26^^,^^28^^,^^29^^,^^33^^,^^34^^,^^35^ Brazier et al. measured the tibial slope in 83 knees using lateral radiographs, and concluded that the methods based on the proximal tibial anatomical axis or the posterior tibial cortex gave higher reliability than other methods did.^29^

In a study on cadavers, Giffin et al. showed that the average increase in tibial slope was 4°, following medial opening wedge tibial osteotomy.^25^ Other authors have reported similar findings.^23^^,^^36^ In our study, the tibial slope presented a mean increase of 2.38°, with a 95% CI ranging from 1.65° to 3.11°. Just as an increase in the slope has been found following medial opening wedge tibial osteotomy, the opposite effect has been demonstrated following closing wedge tibial osteotomy.^23^ The triangular shape of the tibia, with its apex directed anteriorly, may be responsible for an anteromedial opening wedge to create a flexion wedge, and for the opposite to occur in a closing wedge in which more bone is taken anteriorly and subsequent compression leads to a reduction in the tibial slope. Noyes et al. used three-dimensional geometric analysis on the proximal tibia to demonstrate how the opening wedge along the anteromedial cortex influences the tibial slope and valgus correction during osteotomy. They determined that the anterior osteotomy gap at the tibial tubercle needed to be half of the posteromedial gap in order to maintain the normal sagittal tibial slope.^28^

To the best of our knowledge, the correlation between the degree of correction obtained in the frontal plane and the change in the sagittal plane (tibial slope) in medial opening wedge osteotomy has only been examined in a few clinical studies. In this study, we were able to determine that the correction to the front plane (femorotibial angle) and tibial slope (P > 0.05) following opening wedge osteotomy did not have any significant effect.

Agneskirchner et al. demonstrated that opening wedge tibial flexion osteotomy not only caused an increase in the slope, but also resulted in significant anterior tibial translation, thus leading to an anterior shift in the tibiofemoral contact area and decompression of the posterior femoral condyle in an ACL-intact knee.^37^ This decompression has been proposed as a possible treatment option for posterior cartilage damage.^37^ However, no studies have shown the in vivo consequences of anterior contact pressure shifts on cartilage. Giffin et al. evaluated the effects of modifying the tibial slope on knee biomechanics, and observed that small changes to the slope did not result in significant in situ forces in the cruciate ligaments.^25^ However, only a small increase in slope was analyzed, and thus, no predictions were made regarding larger changes in slope. Moreover, the integrity of the meniscus was maintained. Increased in situ forces on the ACL (ranging from 33 to 50%) have been correlated with medial meniscectomy.^38^ The meniscus increases the knee slope by an average of 6°.^39^ This contribution to slope is frequently lost in these patients due to medial meniscectomy. The sum of the effects of the anterior pressure shift, possible increased in situ forces on the ACL and meniscal slope loss is unpredictable, thus making early ACL rupture a plausible hypothesis. The influence of increasing and decreasing the slope and the contribution of the meniscal slope towards the biomechanics of arthritic knees need to be considered for further analysis.

No clinical studies on high tibial osteotomy have demonstrated any statistically better clinical result caused by an increase in tibial slope. In our study, no statistically significant correlation was observed between increased slope and short-term clinical results according to the Lysholm knee score. Yet we have to be cautious with this result, since the population involved only presented a small increase in slope, and the follow-up was short (mean of 40.8 months), before we could observe an evident deterioration of the results with time.

Hernigou et al. reviewed series of patients who underwent medial opening wedge osteotomy and observed that preoperative bone loss in the posterior third of the medial plateau was associated with a slope ranging from 18° to 25°, while a bone loss in the medial third was associated with a slope ranging from 10° to 15°.^40^ Slope was measured using Moore’s technique (plane of the tibial plateau and plane of the anterior crest). In that study at follow-up, patients with bone loss in the posterior third of the medial plateau had a preoperative slope of 16° to 30°, with exception of one patient who developed a 20° slope during operation, which led to anterior subluxation. Both extension and valgus osteotomy have been suggested in cases of excessive slope, since osteotomy did not halt the progress of arthritis.^41^ The biomechanical effects of extension osteotomy on pressure distribution and ligament tension still need to be studied. Likewise, it needs to be determined which cases might benefit from extension osteotomy.

Bonnin suggested that special attention should be given to the tibial slope in ACL-deficient knees, in which there is a direct relationship between the slope and anterior tibial translation.^41^ He demonstrated that for every 10° increase in tibial slope, there is a corresponding 6 mm anterior translation of the tibia. Excessive wear on the posteromedial compartment has been a concern in relation to chronic ACL-deficient knees.^31^^,^^42^ Dejour et al. recommended decreasing the tibial slope in ACL-deficient knees if it exceeded 10°.^24^^,^^41^ Rodner et al. demonstrated that increasing the tibial slope in ACL-deficient knees by means of high tibial osteotomy redistributed the pressure to the posterior tibial slope, and could potentially contribute towards early clinical failure.^42^ Increasing the tibial slope may be beneficial with regard to reducing tibial translation in PCL-deficient knees, whereas decreasing the slope may be protective in ACL-deficient knees.^25^ We did not include unstable knees in our study, but we believe that an increased slope may be a predisposing factor for ACL rupture, and that varus ACL-deficient knees could benefit from osteotomy that maintains or even decreases the tibial slope, thereby avoiding pressure redistribution to the posterior tibial slope and future failure of ACL reconstruction. This raises the question of whether arthritic knees with posterior erosion could function biomechanically as ACL-deficient knees and might also benefit from a decrease in slope.

Significantly greater quadriceps strength required for full knee extension has been correlated with increased slope.^37^ We believe that this can plausibly be explained by the loading on the patellofemoral compartment, which compromises the results in patients who already have patellofemoral osteoarthritis. Increasing or decreasing the normal tibial slope could cause a loss of knee extension or an increase in knee hyperextension, respectively.^28^ Our patients were not evaluated regarding greater strength requirements for extending their knees, but if the above is true, it probably did not affect knee extension. No significant correlation was found between increased slope and loss of extension (P = 0.93). This result reflects the small mean increase in tibial slope (mean of 2.38°), and greater increase in slope might have caused loss of extension.

## CONCLUSION

Medial opening wedge tibial osteotomy led to a small increase in tibial slope. No significant correlation was found between increased tibial slope and short-term clinical results following high tibial osteotomy. Further clinical studies are needed in order to establish whether extension or flexion osteotomy might benefit patients with medial compartment gonarthrosis.
